# The emerging need for family-centric initiatives for obtaining consent in personal genome research

**DOI:** 10.1186/s13073-014-0118-y

**Published:** 2014-12-17

**Authors:** Jusaku Minari, Harriet Teare, Colin Mitchell, Jane Kaye, Kazuto Kato

**Affiliations:** Department of Biomedical Ethics and Public Policy, Graduate School of Medicine, Osaka University, 2-2 Yamadaoka, Suita, Osaka 565-0871 Japan; Centre for Health, Law and Emerging Technologies (HeLEX), Nuffield Department of Population Health, University of Oxford, Old Road Campus, Oxford, OX3 7LF UK; Institute for Integrated Cell-Material Sciences (iCeMS), Kyoto University Yoshida Ushinomiya-cho, Sakyo-ku, Kyoto 606-8501 Japan

## Abstract

The use of information and communication technology can offer a novel way to promote family-centric initiatives for informed consent, and can address associated ethical challenges in personal genome research.

There has been considerable debate on informed consent in personal genome research. This includes, amongst other things, the limitations of broad consent, the difficulty of informed assent (for children to express their willingness for research participation) and proxy consent, and issues of consent for the creation and subsequent use of cell lines such as HeLa cells [1–3]. The use of information and communication technology (ICT) has been suggested as one method to address some of these ethical challenges, through an approach called ‘dynamic consent’, which differs from conventional static consent by enabling research participants to revisit their consent decisions [4,5]. However, while dynamic consent mostly focuses on the relationship between the researchers (or physicians) and research participants, there has been less attention given to its application to family-centric initiatives (FCIs). Consideration of so-called family consent may shed light on ways to address some of the developing ethical issues that are relevant to personal genomic research.

## The relevance of the family in genomic research

The central motivation for considering an FCI approach for informed consent is the anxiety and subsequent debate related to the return of clinically significant findings, including ‘incidental findings’ obtained from research [6,7]. As personal genome research is increasingly concerned with whole genome sequence information, the findings are potentially extensive and could have significant impact for the research participant if disclosed. As an individual’s genomic information is shared with family members, the disclosure of those findings to research participants, in some cases, would also have a considerable influence on their families, specifically their blood relatives [8]. As shown by the concept of ‘mutuality’, which regards a family ‘as a form of pooling and of spreading all known risks’ [9], this family relationship can indirectly transform family members into secondary research participants, or stakeholders. Therefore, it is necessary to consider developing a framework for decision-making that addresses mutuality, and the associated ethical ramifications.

## The challenges of gaining informed consent from family members

Compared with clinical settings [10], FCIs have not really been considered and applied routinely in genomics research. There are five key reasons for this. First, there is the overriding belief that autonomy of the individual is central to informed consent in genome research and the decision whether to consent preferentially therefore rests with the individual. As a result, the conventional informed consent model is not designed to consider the interests of different family members. Second, informed consent procedures are not designed to capture the views or preferences, or to record the decisions of other family members, as they usually involve gaining the individual’s signature at the start of research. This limits how much informed consent procedures can reflect the preferences of family members, who may not be present when the consent form is signed, as there is currently no requirement to consult other family members. Third, the static nature of a paper consent form does not reflect the complexity of decision-making that is required for genomics, when findings may emerge over time as the research progresses. Fourth, there is the perceived practical and logistical burden of introducing the need to contact and communicate with several family members, some of whom may not know about the research study. Fifth, new tools are now available through ICT (particularly web 2.0) that can facilitate social interaction and shared decision-making.

## Family-centric initiatives using ICT

To address these challenges, rapid developments in ICT could be harnessed to facilitate FCIs for consent in personal genome research. Using devices connected to the web, individual research participants and their family members could express their own views on research participation of other family members in advance and also in real time as the research progresses. This approach could provide the opportunity for individuals to make an informed choice on disclosure of research findings, including clinically relevant incidental findings, and would enable primary and secondary research participants to hold different views on this. This is because an FCI approach based on dynamic consent using ICT would not only have the capacity to flexibly manage various options depending on different research contexts and personal preferences, but would also provide the means for families to share and communicate about the findings. A possible outcome would be that this approach could facilitate the promotion of genome research, by creating better relationships between stakeholders, particularly as the relationship between research and clinical settings is becoming increasingly blurred in the area of personal genome research.

## Challenges for implementation

There are several key challenges to enabling the practice of a FCI approach using ICT. One is the definition of the family; it is almost impossible to find a simple answer across the world, because of the diversity of values regarding a family. Another challenge is how to deal with conflicting wishes of family members, how different family members interact with one another, and any associated hierarchies or fractious relationships that may be exacerbated within the research context. In some cases, in terms of privacy and autonomy, the FCI approach could undermine the right for research participants to participate in research projects and to control their own genome information if their families’ interests were considered. It also raises the possibility of coercion - the research project would need to be managed in such a way as to allow family members to express their views confidentially without having to disclose to their relatives whether or not they had agreed to participate.

The introduction of ICT could address many of the logistical difficulties associated with FCIs, and thus provide a platform to identify some of the more complex and emotional issues. By providing effective methods to convey a range of information regarding research projects in a variety of formats, this may help to collect opinions and ease conflicts between family members. Therefore, in designing an ICT system before the implementation of a research project, the identification of relevant family members and the establishment of procedures for their decision-making, incorporating conventional careful face-to-face communication, would play a crucial role in the effectiveness of FCIs. Bespoke design would also enable different cultural norms and the specificities of social contexts to be taken into account.

Ultimately, novel and alternative approaches, moving away from traditional, front-loaded informed consent, have the potential to further facilitate research progress and promote public trust in research. They can also address the emerging importance of the family in personal genome research, due to an increased awareness of clinically significant findings and the unique nature of genome information shared between family members. By providing a direct communication channel it will be easier to consider the views of family members who could be affected by the return of incidental research findings. We anticipate that the FCI approach would not necessarily be appropriate for all research projects, but it would have significant benefits for broad research contexts, including family-based study, pediatric research and genome cohorts, such as a three-generation cohort study.

## Conclusions

A FCI approach using ICT may raise new technical, financial and social challenges, but it would also deliver novel benefits, through dynamic communication, for several stakeholders, including research participants and their families, researchers, ethics review committees and genetic counsellors. The feasibility of the FCI-based consent with ICT can be reinforced by the possibilities of introducing digital interfaces to facilitate digital or e-governance in biomedical research. It might be applicable not only to other biomedical research but also more widely in the clinic, including for genetic testing, organ donation and cancer notification.

For FCI consent to work using a dynamic consent approach, it could require engagement of ICT across different generations in one family. It will be important to ensure that mechanisms are in place for individuals who are not familiar with, or comfortable using, ICT to contribute their views. Further studies that explore the use of ICT across generations, and that recognize cultural differences and can learn from diverse approaches to the family, will help to further explore and develop the possibility of applying an FCI approach usefully in biomedical research.

## Abbreviations

FCI: Family-centric initiative
ICT: Information and communication technology
